# Discussion on Dr. Parker’s Paper—Enteritis

**Published:** 1892-01

**Authors:** 


### GENERAL DISCUSSION OF PAPERS READ.

DISCUSSION ON DR. PARKER'S PAPER CONTINUED*.—Dr. J. M. Frazer, of Morgan, said: I think no physician, situated as Dr. Parker was, could have made a better diagnosis from the symptoms. The scarcity of literature on the subject is unfortunate. Even in Wood's Medical History of the War no reference is made to any case similar to the one just reported. I have seen no case presenting such symptoms, but I can conceive how a circumscribed inflammation could bring about a destruction of peristalsis, thus producing symptoms like obstruction. The symptoms enumerated in Dr. Parker's paper point to a grave condition, and I think, under any treatment the patient would have died; recovery would have been possible, perhaps, if the case had been seen earlier, and a correct diagnosis made while it was possible. As to the distinctions of terms, typhilitis, peri-

[*Case of Enteritis Simulating Obstruction of Bowels, by Daniel Parker, M. D., published in the November number of this JOURNAL.]

typhilitis, etc., we all know that the inflammation area does extend to adjacent parts, and it is not always possible to accurately locate the lesion in all cases. The general character indicates, however, if the lesion be in the colon, or in the small intestine. I agree with the writer and am opposed to the do nothing plan. Exploration should be made in urgent cases. I will relate a case illustrating my position.

I had a patient suffering from obstinate constipation which medicine would not relieve,—a man who could speak very little English, and I could not get a perfect history. He had an irreducible right inguinal hernia—the bowel down in scrotum. He had also a hydrocele, which, with the bowel, greatly distended the bag and I feared strangulation. There was no evidence of inflammation rendering operation urgent and I waited; used enemas, but they came away unchanged. Meantime—second day —stercoraceous vomiting set in, and I was sure there was strangulation, and prepared to operate; he was about to go into a state of collapse, and, preparatory to operating, gave another enema, which, to my surprise and his relief, brought away scybaläe and afterwards a free fecal discharge. Hernia, however, remained unrelieved. This shows we should not be in a hurry to open the abdomen. Too many surgeons are like Alexander, they want to conquer—with the knife. A patient and careful study of symptoms will often enable us to diagnose correctly. I agree with Dr. Parker as to the value of opium in these cases.

Prof. H. A. West said, the idea of complete obstruction of the bowel without the presence of a foreign body, obstruction from paresis alone, was something new to him. Has seen cases of retained undigested food, with evolution of gases, and spasmodic contraction of bowels, as in ordinary colic. Where there is entire absence of foreign body, obstruction by paresis alone is rare. Know no literature on the subject. But no doubt they do occur. In case of obstruction from spasm of bowel, opium will relieve the patient. Obstruction by fecal impaction is a very different thing; but differential diagnosis between obstruction from spasm, from feces, from foreign bodies and other causes, is not always easy. As to operating, we can be too conservative,—this thing of not invading the "sacred precincts" can be overdone. In uncertain diagnosis, death threatening, case not at all clear, and you think operation affords reasonable hopes—laparotomy is justifiable. And where ordinary means have failed to overcome the obstruction, from whatever cause, an exploratory operation should be made. Unless we operate we know these cases die, and too often the operation is deferred too long.

Prof. J. E. Thompson, Texas Medical College, said: The theory of paralysis of a portion of bowel acting as a complete obstruction is quite tenable. Any surgeon who has experience in operations for strangulated hernia, knows that the cause of after symptoms of strangulation, persisting even after the relief of constriction, is often due to complete paralysis of the knuckle of bowel after reduction. I would remark on the fact that there was a certain transition from the inflamed to the non-inflamed bowel, and I regret that the author has not mentioned definitely, whether or no he had looked for a possible cause of strangulation, such as an omental band. Such strangulations occur and are often relieved spontaneously. I recall a case where I performed a post mortem examination on a man who had died from the effects of an overdose of morphia. In addition to the signs of morphia poisoning I found a loop of the small intestines, about six inches long, acutely congested. The line of demarcation between this and the healthy bowel was well marked. On searching farther I found an omental band stretching from the lower part of the great omentum to the anterior abdominal wall, to which it was firmly attached. This formed a bridge, under which the bowel had passed and under which it had probably been constricted. The evidence in court showed that the patient had been subject to repeated attacks of colic and had been in the habit of taking morphia to relieve the pain.

I congratulate the writer on the masterly way he has treated the differential diagnosis. I would also point out that abdominal section is not to be condemned in these cases as long as the surgeon has enough common sense to refrain from doing too much.

Prof. Thompson exhibited two pathological specimens as follows:

1. A larynx, showing a vertical cut through the thyroid cartilage dividing it into two halves.

This was the result of an attempted suicide in a man suffering from delirium during an attack of typhoid fever. This case was interesting from the extreme rarity of such cases, showing a vertical division of the thyroid cartillage.

2. An enlarged bursa, filled with and its walls infiltrated with urate of soda.

This was removed from over the olecranon process in a man who suffered from gout, and who had manifestations of gout at that time, viz: tophi, gouty inflammation of the phalangeal joints, a ganglion at the wrist, connected with the tendon of the extensor carpi ulnaris and which was filled with the urate of soda. The case was also interesting from the association of the gouty phenomena with typical and rheumatoid arthritis of the joints of the fingers and knees.

